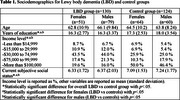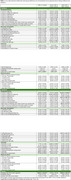# Sex‐Specific Environmental and Occupational Risk Factors for Lewy Body Dementia

**DOI:** 10.1002/alz70860_106129

**Published:** 2025-12-23

**Authors:** Francesca R. Dino, Danelle J. Carter, Reilly Costello, Hamidreza Ghodsi, Charlotte S. Rivera, Irene Litvan, Ece Bayram

**Affiliations:** ^1^ University of Colorado, Anschutz Medical Campus, Aurora, CO, USA; ^2^ University of California San Diego, La Jolla, CA, USA; ^3^ University of California, San Diego, La Jolla, CA, USA; ^4^ University of Colorado Denver, Aurora, CO, USA

## Abstract

**Background:**

Lewy body dementia (LBD) risk differs by sex, with males at higher risk than females. Environmental and occupational exposures throughout life also vary by sex and may contribute to this difference. Despite the identification of various environmental and occupational risk factors in Alzheimer's and Parkinson's diseases, research on LBD remains limited.

**Method:**

We included data from 130 individuals with LBD (51 females, 79 males) and 124 controls of similar age (64 females, 60 males) from LBD‐TOROS, a remote cross‐sectional survey study. Environmental and occupational factors during childhood and adulthood, including residential history, beverage consumption (e.g. caffeinated drinks), toxicant exposure, physical activity, smoking, and alcohol use, were compared between groups using Chi square and Mann‐Whitney U tests. Associations between significantly differing variables and LBD risk were assessed with logistic regression. Analyses were conducted for the overall cohort and sex‐stratified cohorts.

**Result:**

The majority of the cohort identified as cisgender and straight (94.1%), and non‐Hispanic and White (75.2%). Individuals with LBD had lower years of education, lower income, and lower subjective social status than controls (Table 1). Compared to controls, individuals with LBD had a higher prevalence of exposure to metal dust/dust fumes in childhood and in adulthood, glue/adhesive at work, welding/brazing/flame cutting metal, soldering, and smoking (Table 2). In the overall cohort, smoking, not living in a suburb, exposure to glue/adhesive at work, welding/brazing/flame cutting metal, soldering, and metal dust/dust fumes in adulthood were significantly associated with LBD risk. For females, decaffeinated coffee, caffeine‐free soda during childhood, not living in a suburb, and exposure to pesticides at work in adulthood were associated with LBD risk. For males, not living in a large city, private well water, caffeinated green tea, welding/brazing/flame cutting metal, soldering, metal dust/dust fumes in adulthood, and smoking were associated with LBD risk.

**Conclusion:**

Environmental and occupational factors in both childhood and adulthood can increase LBD risk. Identifying sex‐specific risk factors can provide insights into disease pathogenesis and guide effective prevention and management strategies. However, current LBD literature stems mostly from male participants, and more research is needed on LBD in females.